# Hemophagocytic lymphohistiocytosis directly triggered by peginterferon alfa-2b in a patient with chronic hepatitis B

**DOI:** 10.3389/fimmu.2026.1760610

**Published:** 2026-02-27

**Authors:** Peipei Wang, Jiahui Pang, Huiming Xu, Menglan Wang, Wenxing Lai, Dayang Hui, Qingxian Cai, Xudong Li, Jianyun Zhu

**Affiliations:** 1 Department of Infectious Diseases, The Third Affiliated Hospital of Sun Yat-sen University, Guangzhou, Guangdong, China; 2 Department of Hematology, The Third Affiliated Hospital of Sun Yat-sen University, Guangzhou, Guangdong, China; 3 Department of Pathology, The Third Affiliated Hospital of Sun Yat-sen University, Guangzhou, Guangdong, China; 4 Department of Infectious Diseases, Shenzhen Third People’s Hospital, Shenzhen, Guangdong, China

**Keywords:** chronic hepatitis B, hemophagocytic lymphohistiocytosis, opportunistic infections, peginterferon alfa-2b, soluble interleukin-2 receptor

## Abstract

This case report describes a 42-year-old male with chronic hepatitis B (CHB) who developed hemophagocytic lymphohistiocytosis (HLH) following treatment with peginterferon alfa-2b (PegIFN-α-2b). The patient tolerated the initial injections well. After the 16th injection in February 2025, laboratory tests revealed cytopenia, prompting discontinuation of PegIFN-α-2b. The onset of a high-grade fever approximately three weeks after drug cessation coincided with the timeframe for the drug’s clearance, suggesting a continued immunostimulatory effect. HLH was diagnosed based on hyperferritinemia (>50,000 ng/mL), elevated soluble interleukin-2 receptor (sCD25), and hemophagocytosis on bone marrow biopsy. He responded well to etoposide and dexamethasone. However, his course was complicated by sequential opportunistic infections: severe anemia due to parvovirus B19 (confirmed by plasma metagenomic next-generation sequencing, mNGS) and subsequent herpes simplex virus (HSV) encephalitis (diagnosed via CSF mNGS). Both complications were managed successfully with intravenous immunoglobulin and acyclovir, respectively. This case highlights PegIFN-α-2b as a rare direct trigger of HLH in CHB and underscores the critical risk of opportunistic infections during immunosuppressive therapy, demonstrating the pivotal role of mNGS in diagnosing elusive infections in immunocompromised hosts.

## 1 Introduction

Hemophagocytic lymphohistiocytosis (HLH) is a life-threatening hyperinflammatory syndrome characterized by the uncontrolled activation of cytotoxic T lymphocytes and macrophages, resulting in a cytokine storm and immune-mediated organ damage (1). HLH is historically classified into primary (familial) HLH, caused by genetic mutations impairing cytotoxic function, and secondary (acquired) HLH, which occurs in the absence of a known genetic defect (2).

Secondary HLH is triggered by strong immunological stimuli. The most common triggers are infections, particularly viral (Epstein-Barr virus, EBV; Cytomegalovirus, CMV; Human Immunodeficiency Virus, HIV), followed by bacterial (e.g., *Mycobacterium tuberculosis*), parasitic, and tick-borne infections (3, 4). Other major categories include malignancy-associated HLH (predominantly lymphomas) and autoimmune-associated HLH (macrophage activation syndrome) (4). Although less common, drug-induced HLH is a recognized entity, with agents such as anticonvulsants, sulfonamides, and immunotherapies implicated (5).

Interferon-alfa (IFN-α) is a cornerstone therapy for Chronic Hepatitis B (CHB), aimed at achieving functional cure via immune modulation (6). While its common side effects (cytopenias, flu-like symptoms) are well-known, its potential to trigger severe dysregulated immunity like HLH is exceptionally rare. To our knowledge, no case has been reported where IFN-α therapy directly triggered HLH in a CHB patient without an intervening autoimmune disorder like SLE (7). Here, we report a case of HLH directly induced by Peginterferon alfa-2b (PegIFN-α-2b) in a CHB patient, further complicated by sequential opportunistic infections (Parvovirus B19 and HSV-1 encephalitis), highlighting a particularly challenging scenario in managing the balance between immunosuppression and infection risk.

## 2 Case presentation

### 2.1 History and initial treatment

A 42-year-old male with a 20-year history of chronic hepatitis B virus (HBV) infection presented with recurrent high fever on March 11, 2025. He had been treatment-naïve until September 2024, when elevated alanine aminotransferase (ALT) and aspartate aminotransferase (AST) prompted the initiation of combination therapy: tenofovir alafenamide (TAF, 25 mg daily) and peginterferon alfa-2b (PegIFN-α-2b, 180 μg weekly). Pre-treatment screening, including complete blood count and autoimmune antibodies was unremarkable. The patient tolerated the regimen well initially. However, after the 16th PegIFN-α-2b injection on February 10, 2025, routine monitoring revealed leukopenia and thrombocytopenia. Consequently, PegIFN-α-2b was discontinued, while TAF monotherapy was maintained.

### 2.2 Onset of HLH

Three weeks post-discontinuation of PegIFN-α-2b (March 2, 2025), the patient developed a high fever (peak temperature of 40 °C) with mild cough. He was admitted to a local hospital on March 7. Initial workup, including chest CT and echocardiogram, was unremarkable. Abdominal ultrasound revealed moderate liver fat and mild spleen enlargement. Lab results showed high AST (954 U/L), ALT (595 U/L), and pancytopenia (WBC 2.97 × 10^9^/L, PLT 114 × 10^9^/L). Due to the flu season, he received empiric oseltamivir, alongside moxifloxacin and meropenem; however, influenza antigen testing was negative. His condition deteriorated, with worsening pancytopenia and transaminitis, prompting transfer to our center on March 11.

### 2.3 Diagnosis and HLH management

**Figure 1:**
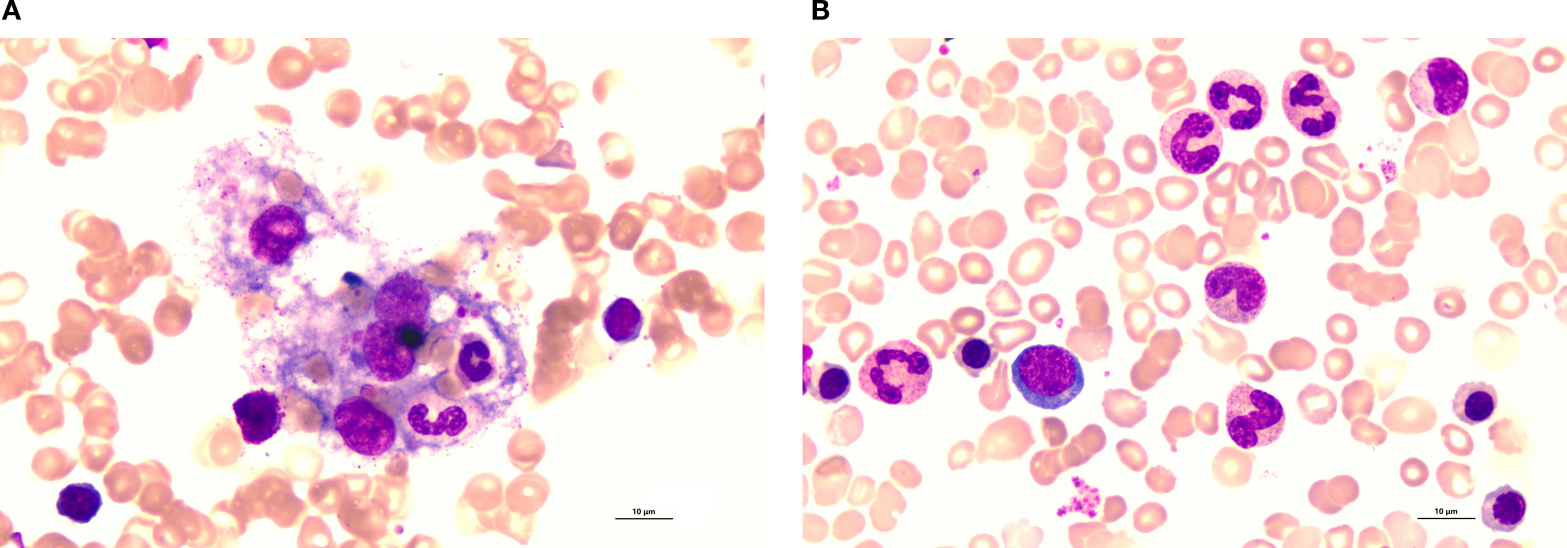
Bone marrow biopsy findings at diagnosis and after treatment. **(A)** Bone marrow biopsy at initial diagnosis (March 12) shows characteristic hemophagocytosis. **(B)** A follow-up bone marrow biopsy after treatment (April 18) shows resolution of hemophagocytic activity with normal hematopoietic cells.

Upon admission, the patient was febrile (39.1 °C), tachycardic (heart rate 142 bpm), and tachypneic (respiratory rate 24 breaths/min). Physical examination was unremarkable except for hepatosplenomegaly confirmed by ultrasound (liver right lobe 139 mm and spleen length 131 mm). Laboratory evaluation (**Table 1**) revealed severe pancytopenia, hyperferritinemia (>50,000 ng/mL), and elevated soluble CD25 (11,207 U/mL). Crucially, hepatitis B virological markers at this time confirmed effective suppression: HBsAg was positive, HBeAg was negative, anti-HBe was positive, and HBV DNA was undetectable (< 10 IU/mL). An extensive infectious workup (blood cultures, respiratory pathogens, Brucella and initial plasma metagenomic next-generation sequencing, mNGS) was negative. A PET-CT scan showed hepatosplenomegaly with increased metabolic activity in the liver, spleen, and bone marrow. A bone marrow biopsy on March 12 confirmed hemophagocytosis (**Figure 1A**).

**Table 1:** Key laboratory parameters during hospitalization and follow-up.

Parameters	Admission (Mar 11)	After treatment (Mar 20)	Follow-up late Aug 2025	Reference interval
Hematology				
WBC (×10^9^/L)	2.28	3.6	8.03	3.5-9.5
HB (g/L)	109	102	138	130-175
PLT(×10^9^/L)	87	104	205	100-350
Liver function and biochemistry				
ALT((U/L))	477	384	33	3-35
AST((U/L))	1207	548	26	15-40
TBIL (μmol/L)	38.06	35	12.3	4.0-23.9
DBIL (μmol/L)	30.03	25.6	3.9	0-6.8
ALB (g/L)	27.7	31.2	40.5	36-51
CR(mg/L)	45.1	50	54	31.8-116
LDH(U/L)	1608	945	180	71-231
PT(sec)	15.3	17.1	13.3	11.0-14.5
TRIG(mmol/L)	3.47	2.82	1.89	0.34-1.92
Inflammatory markers				
Ferritin (ng/mL)	73934	33511	200.8	21.81-274.66
sCD25(U/mL)	11207	5382	550	223-710
IL-6 (pg/mL)	526.2	20.09	6.18	0-7
PCT(ng/mL)	0.76	0.22	0.02	0-0.05
SAA(mg/L)	47.33	54	–	0-10
CRP(mg/L)	45.1	1.79	1.3	0-6

**Figure 2:**
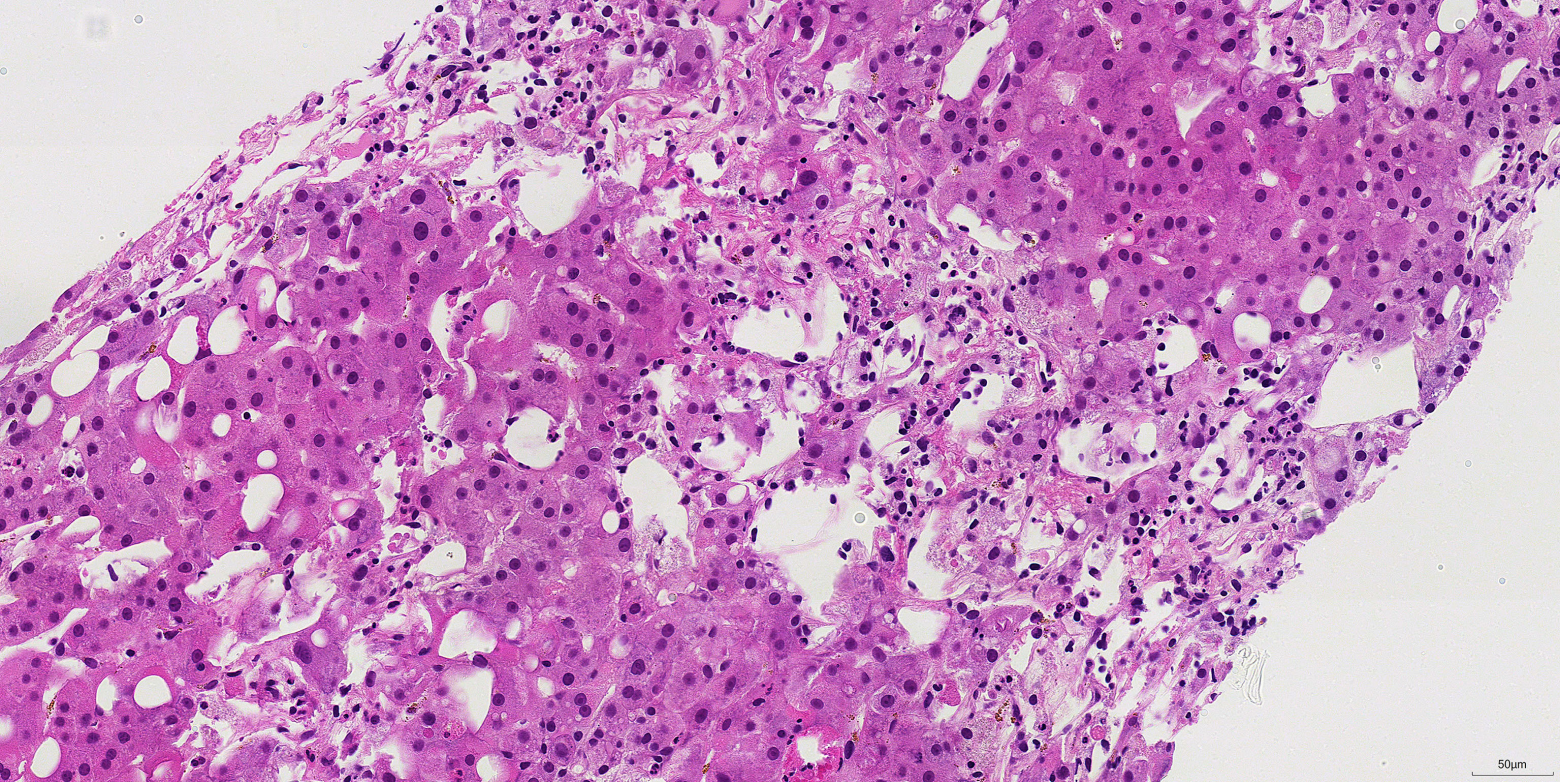
Liver biopsy histology revealing features consistent with HLH-associated liver injury. The micrograph demonstrates patchy hepatocyte necrosis accompanied by a lymphocytic infiltrate. This pattern of injury, which differs from that typically seen in chronic viral hepatitis, is consistent with hepatic involvement in the setting of systemic hyperinflammation (HLH).

HLH was diagnosed, and the patient was started on the HLH-94 protocol (dexamethasone (10 mg/m² IV) and etoposide (100 mg/day IV, twice weekly)) on March 14 after consulting with the hematology department. Fever resolved within 48 hours, and ferritin levels dropped precipitously. A liver biopsy performed on March 20 to investigate residual liver injury revealed hepatocyte necrosis and lymphocytic infiltration consistent with HLH, rather than viral hepatitis (**Figure 2**). By March 31, imaging showed the spleen size had decreased significantly. During chemotherapy, the patient developed a herpes simplex infection near the mouth, which was treated with acyclovir ointment and resolved. The patient improved clinically and was discharged on April 2 for further HLH treatment at a local hospital.

### 2.4 Sequential opportunistic infections

The patient was readmitted on April 17 with severe anemia (Hb 83 g/L) and fever (up to 39 °C). Bone marrow biopsy showed no HLH recurrnce (**Figure 1B**) but finding consistent with pure red cell aplasia, plasma mNGS identified Parvovirus B19. He was diagnosed with parvovirus B19-associated anemia, likely representing reactivation in the setting of immunosuppression. Treatment with intravenous immunoglobulin (IVIG, 0.4 g/kg/day for 5 days) was initiated on April 18, with etoposide temporarily held. He became afebrile on April 19.

Shortly thereafter, on April 21, the patient developed headache and cognitive slowing. Neurological examination was initially normal but later suggested neck stiffness. A lumbar puncture revealed elevated opening pressure. Cerebrospinal fluid (CSF) analysis showed elevated protein. CSF mNGS identified Herpes Simplex Virus type 1 (HSV-1). Brain MRI showed white matter lesions in the left insular and temporal lobes, consistent with encephalitis. A diagnosis of HSV-1 encephalitis was made, and high-dose intravenous acyclovir (500 mg every 8 hours) was started immediately. HLH therapy (etoposide and dexamethasone) was continued in consultation with hematology. Serial lumbar punctures showed gradual normalization of CSF parameters. The patient’s neurological symptoms improved significantly.

**Figure 3:**
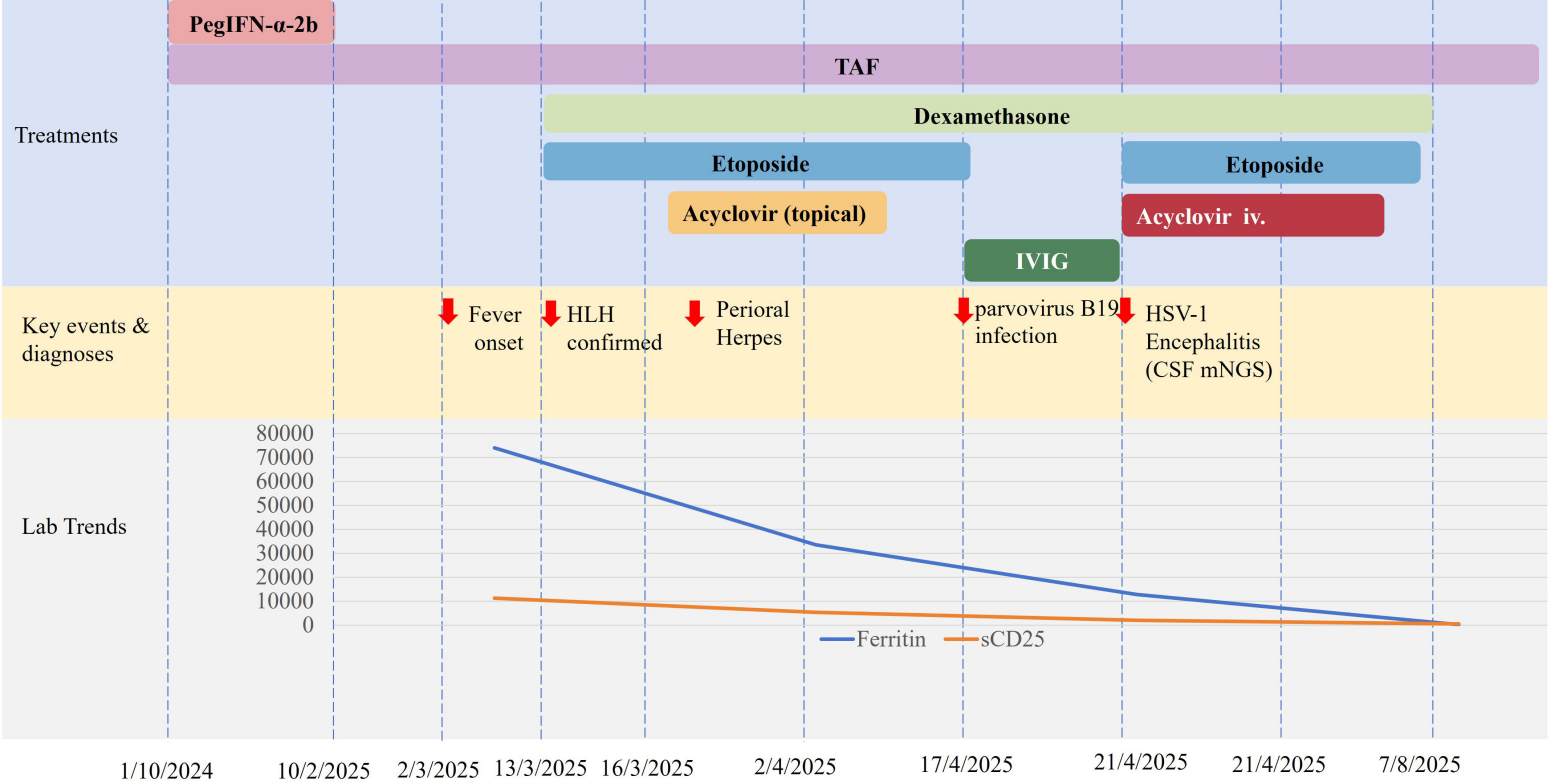
Timeline of clinical events, therapeutic interventions, and key laboratory parameters. The upper section (“Treatments”) illustrates the duration of administered pharmacotherapies. The middle section (“Key events & diagnoses”) marks critical clinical milestones. The lower section (“Lab Trends”) conceptually depicts the trajectory of two pivotal laboratory parameters for Hemophagocytic Lymphohistiocytosis (HLH) diagnosis and monitoring: Ferritin and soluble interleukin-2 receptor (sCD25). The timeline highlights the sequence from peginterferon alfa-2b (PegIFN-α-2b) exposure and discontinuation, to the development and treatment of HLH, and the subsequent occurrence of opportunistic infections (parvovirus B19 and herpes simplex virus type 1 encephalitis) during immunosuppressive therapy.TAF, tenofovir alafenamide; IVIG, intravenous immunoglobulin; HSV-1, herpes simplex virus type 1.

The patient made a full recovery and returned to work by August 2025 and the clinical course is summarized in **Figure 3**.

## 3 Discussion

This report presents a rare and instructive case of PegIFN-α-2b directly triggering HLH in a patient with CHB, which was further complicated by life-threatening opportunistic infections during immunosuppressive therapy. The case underscores the delicate balance between controlling a hyperinflammatory state and preserving host immunity, while highlighting the critical role of advanced diagnostic technologies. The successful outcome underscores the importance of vigilant monitoring, advanced diagnostic techniques, and a multidisciplinary approach in managing such critically ill patients.

Establishing the trigger is vital. We identified PegIFN-α-2b as the culprit based on temporal association and exclusion of alternatives. The onset occurred 3 weeks after PegIFN cessation. This aligns with the long half-life of PegIFN, which can exert immunomodulatory effects for weeks (8), creating a “cytokine storm” environment even after drug withdrawal (9). Other potential triggers were systematically excluded. HBV-associated HLH usually occurs during high viral replication (10). Our patient had undetectable HBV DNA due to effective TAF suppression. Furthermore, the liver biopsy showed confluent necrosis and lymphocytic infiltration typical of HLH/DILI, distinct from the piecemeal necrosis and ground-glass hepatocytes seen in active CHB (11, 12). The hepatic injury observed in the biopsy (March 20) significantly predated the onset of Parvovirus B19 viremia (April 17), arguing against Parvovirus as the cause of the liver pathology. TAF itself lacks significant immunomodulatory activity and was continued throughout the HLH crisis and recovery (13). Had TAF been the trigger, the disease would likely have persisted. Additionally, TAF is not a known trigger for HLH in current literature. Thorough investigation ruled out other common secondary causes like malignancy or active infection with typical HLH-associated pathogens (e.g., EBV, CMV) at presentation (2, 3). To our knowledge, this is the first reported case of HLH directly induced by IFN-α therapy in a CHB patient without an intervening autoimmune disorder.

This case highlights the challenge of managing HLH: the need to control a life-threatening cytokine storm with immunosuppressive therapy, which weakens the body’s defenses against infections (14). Our patient’s experience exemplified this dilemma, resulting in two consecutive viral complications. The development of localized perioral herpes during initial chemotherapy was a critical warning sign. In immunocompromised hosts, mucocutaneous herpes simplex virus (HSV) infection should not be viewed as a benign complication but as an indicator of systemic reactivation risk (15). The subsequent diagnosis of HSV-1 encephalitis, likely due to neuronal spread from reactivated latent virus in the setting of T-cell immunosuppression, underscores this point. This suggests that the appearance of such lesions in similar patients warrants not only local therapy but strong consideration of systemic antiviral prophylaxis to prevent catastrophic central nervous system dissemination (16). The subsequent parvovirus B19 infection, causing pure red cell aplasia, is a well-documented complication in immunocompromised adults due to the virus’s tropism for erythroid progenitor cells (17).

For secondary HLH with an identified and removable trigger, such as a drug, therapy aims to control hyperinflammation while facilitating trigger removal (18). The HLH-2004 protocol (etoposide/dexamethasone) remains standard for its proven efficacy in rapidly abating the hyperinflammatory state (19). The emergence of targeted biologics (e.g., anakinra, emapalumab) offers promise for more precise immunomodulation with potentially fewer infectious complications (20). The prognosis of HLH varies significantly by trigger. Infection-associated HLH, particularly with viruses like EBV, carries high mortality if untreated (21). Bacterial and tick-borne triggers also confer substantial risk (22, 23). In contrast, drug-induced HLH often has a more favorable outcome if the offending agent is withdrawn and immunosuppression is initiated promptly (24). This underscores the prognostic importance of meticulous trigger identification.

This case serves as a powerful testament to the value of mNGS in managing complex, immunocompromised patients (25). Conventional, targeted microbiological tests repeatedly returned negative results, leaving the team without a diagnosis at critical junctures. The unbiased nature of plasma mNGS allowed for the rapid and unexpected identification of parvovirus B19, directly guiding successful treatment with IVIG. More critically, mNGS of the cerebrospinal fluid provided a life-saving diagnosis of HSV-1 encephalitis at a stage when clinical symptoms were non-specific and CSF abnormalities were mild. This enabled the immediate initiation of high-dose acyclovir, an intervention where delays are directly linked to poor neurological outcomes (26, 27). This case strongly supports the early deployment of mNGS in immunocompromised hosts with unexplained febrile syndromes or neurological decline, as it can circumvent diagnostic biases and reveal unexpected pathogens (28).

In summary, PegIFN-α-2b is a rare but potent trigger for HLH. Clinicians must distinguish this from viral flares or sepsis. Furthermore, the necessary treatment for HLH imposes a severe risk of opportunistic infections, necessitating vigilant monitoring and a low threshold for advanced diagnostics like mNGS.

## Data Availability

The raw data supporting the conclusions of this article will be made available by the authors, without undue reservation.
